# Sex differences in *in vivo* biomarkers of neurodegenerative dementia

**DOI:** 10.3389/frdem.2025.1706177

**Published:** 2025-12-09

**Authors:** Elijah Mak, Kejal Kantarci, Zoe Arvanitakis

**Affiliations:** 1 Department of Radiology, Mayo Clinic, Rochester, MN, United States; 2 Rush Alzheimer’s Disease Center, Department of Neurological Sciences, Rush University, Chicago, IL, United States

**Keywords:** dementia, Lewy bodies (LBD), neuroimaging, biomarker, sex differences, Alzheimer

## Abstract

**Background:**

Alzheimer’s disease (AD) and dementia with Lewy bodies (DLB) are two of the most common neurodegenerative diseases in older adults, and both show well-documented sex-specific differences in terms of clinical presentation, prevalence, and progression trajectories. However, the underlying neurobiological substrates that underpin these differences are poorly understood. *In vivo* biomarkers are well-suited to yield insights into how biological sex may shape disease pathophysiology in AD and DLB, and thus inform future research and precision medicine. The objective of this review is to synthesize recent evidence on sex differences related to biomarkers of AD and DLB.

**Methods:**

We conducted a literature search of PubMed for studies published between January 2000 and May 2025 examining sex differences in neuroimaging and biofluid markers of mild cognitive impairment (MCI), AD, and/or DLB. Eligible studies were required to include sex-stratified or sex-interaction analyses in human participants with clinically defined MCI (due to AD or DLB), AD, or DLB.

**Results:**

Of a total of 261 articles imported for screening, 63 met inclusion criteria, comprising 50 cross-sectional and 13 longitudinal investigations across biofluid markers (*n =* 18) studies, structural imaging (*n =* 18), functional imaging (*n =* 16), and molecular imaging (*n =* 11) studies. Women demonstrated initial cortical structural and metabolic advantages followed by accelerated decline. In MCI and AD, women were generally more susceptible to tau pathology and APOE ε4-related risk. In contrast, men with DLB showed greater metabolic and dopaminergic abnormalities, though women with DLB frequently exhibited mixed biomarker profiles. APOE ε4 conferred increased vulnerability in women for both conditions. Biofluid markers also revealed sex-specific expression patterns and associations with clinical outcomes.

**Discussion:**

There is growing evidence that biological sex significantly influences the pathophysiology of AD and DLB as captured by *in vivo* biomarkers. These findings highlight the growing importance of analyses that consider sex differences in biomarker research and support the development of personalized diagnostic and therapeutic strategies in neurodegeneration. Future research should prioritize longitudinal studies to define optimal biomarker sequencing and therapeutic windows for each sex, while also investigating the genetic, hormonal, metabolic, pharmacological, and environmental mechanisms that underlie these sex differences, ultimately advancing precision medicine in neurodegenerative disease.

## Introduction

Dementia affects over 55 million people worldwide, with Alzheimer’s disease (AD) and dementia with Lewy bodies (DLB) representing two of the most common and clinically recognizable neurodegenerative causes, collectively accounting for a significant proportion of age-related dementia cases worldwide. AD affects approximately 60–70% of dementia patients and is characterized by progressive memory decline, amyloid-*β* (Aβ) and tau accumulation, and widespread cortical atrophy (Dubois et al., 2007; Scheltens et al., 2021). DLB represents 10–15% of dementia cases and presents with fluctuating cognition, visual hallucinations, parkinsonian motor symptoms, and is neuropathologically defined by *α*-synuclein pathology (McKeith et al., 2017; Vann Jones and O’Brien, 2013). Despite shared features of progressive cognitive decline, both AD and DLB exhibit distinct pathophysiological signatures that have become increasingly accessible to delineate through advances in the development of neuroimaging and biofluid markers.

The role of neuroimaging and biofluid markers has become central to early diagnosis, especially as the field has moved toward a biologic definition of disease rather than using a purely syndromic approach (Jack et al., 2018). Further, these biomarkers are also critical for disease monitoring and treatment development in both AD and DLB. Briefly, structural MRI reveals distinct patterns of cortical atrophy in AD, particularly in the medial temporal lobe, while DLB demonstrates more pronounced posterior cortical thinning and basal ganglia involvement (Mak et al., 2014). And, more recently positron emission tomography (PET) enables *in vivo* quantification of amyloid and tau deposition in both AD and DLB (Hall et al., 2017; Kantarci et al., 2016; Mak et al., 2021). Emerging neuroimaging techniques, including diffusion tensor imaging (DTI) and functional MRI (fMRI), along with plasma and cerebrospinal fluid (CSF) biomarkers, collectively provide additional insights into network connectivity, metabolic dysfunction, and pathological changes.

Yet, while these biomarkers have significantly improved diagnostic accuracy and differential diagnosis (Hansson et al., 2022; Kantarci et al., 2016), the extent to which their expression differs by biological sex remains a largely underexplored area of investigation. This knowledge gap is particularly striking, given the well-documented clinical and phenotypic differences between men and women in both AD and DLB presentation and progression, and that the biological mechanisms underlying these sex differences remain poorly understood (Carter et al., 2012). Women demonstrate a higher lifetime risk of developing AD, show faster rates of cognitive decline, and exhibit greater tau burden and cortical atrophy (Altmann et al., 2014; Buckley et al., 2019; Oveisgharan et al., 2018). The influence of the APOE ε4 allele, a major genetic risk factor for AD (Corder et al., 1993), also appears more pronounced in women, particularly regarding cognitive decline and associations with tau pathology (Sundermann et al., 2019). In contrast, DLB is diagnosed more frequently in men (Kane et al., 2018), a pattern consistently observed across prior American, European, and Japanese autopsy series that have found disproportionate numbers of men in pathologically-confirmed DLB cohorts (Barker et al., 2002; Fujishiro et al., 2008; Weiner et al., 1996). Men may present earlier with core features such as REM sleep behavior disorder and parkinsonism, while women with DLB tend to show more co-existing AD pathology (Diaz-Galvan et al., 2023) and are more likely to be underdiagnosed (Bayram et al., 2021). Together, these divergent clinical trajectories and presentations suggest that sex-specific biological pathways contribute to the pathophysiology of AD and DLB.

However, current biomarker thresholds and diagnostic frameworks do not account for sex-related variations, potentially limiting early detection and treatment optimization and therefore overlooking insights that could inform more precise diagnostic and therapeutic approaches (Nebel et al., 2018). For instance, a growing body of evidence suggests that sex differences have a bearing on treatment responses in disease-modifying trials, necessitating sex-stratified analyses to optimize therapeutic outcomes (Andrews et al., 2025). The cholinergic system—a primary target for AD and DLB treatments—exhibits marked sexual dimorphism too. Women showed higher frontal cortex cholinergic activity while men demonstrated greater hippocampal cholinergic function, with sex hormones exerting distinct trophic effects on cholinergic neurons through estrogen and androgen receptors (Giacobini and Pepeu, 2018). These neurobiological differences translate to clinically significant treatment disparities, with men showing stronger and more selective benefits from cholinesterase inhibitors in AD, potentially due to sex-specific differences in cholinergic system structure, function, pharmacokinetics, and aging-related changes (Giacobini and Pepeu, 2018).

Our review aims to synthesize current evidence on sex differences in *in vivo* biomarkers of AD and DLB and patients with mild cognitive impairment (MCI) due to AD or DLB. We included studies in MCI because it represents an intermediate stage between normal aging and dementia, marked by measurable cognitive decline with largely preserved daily functioning. Studying sex differences at this stage may reveal early biological mechanisms that precede dementia. By evaluating sex-specific findings from structural, functional, and molecular imaging, as well as biofluid markers modalities, we aim to identify consistent patterns and broad themes that may inform future research directions and support the advancement of personalized therapeutic strategies in AD and DLB.

## Methods

This review was conducted in accordance with the Preferred Reporting Items for Systematic Reviews and Meta-Analyses (PRISMA) guidelines. To identify relevant studies, we conducted a PubMed search limited to articles published in English between January 1, 2020, and May 27, 2025, with a focus on sex-related differences in imaging and biofluid markers of AD, DLB and MCI. The keywords were: ((“sex differences” OR “gender differences” OR “sex dimorphism” OR “sexual dimorphism” OR “sex-specific” OR “sex stratified” OR “sex dependent” OR “sex by” OR “sex X” OR “sex interaction” OR “gender interaction”) AND (“plasma biomarker*” OR “CSF biomarker*” OR “serum biomarker*” OR “blood biomarker*” OR neuroimaging OR MRI OR PET OR SPECT OR “magnetic resonance imaging” OR “positron emission tomography” OR “single-photon emission computed tomography” OR “brain imaging” OR “brain volume*” OR “brain atrophy”) AND (“Alzheimer’s disease” OR “Alzheimer disease” OR “AD” OR “Lewy bod*” OR “DLB” OR “mild cognitive impairment due to Alzheimer*” OR “MCI due to Alzheimer*” OR “Dementia with Lewy bodies”) NOT (animal* OR mice OR mouse OR rat OR rats OR rodent* OR “early onset” OR “early-onset” OR “familial” OR “autosomal dominant” OR “presenilin” OR “healthy control*” OR “healthy adult*” OR “cognitively normal”)) AND (“2020/01/01”[Date - Publication]: “2025/05/27”[Date - Publication]).”

### Eligibility criteria

For inclusion, studies were required to: (1) report original data from human participants with MCI and/or AD and/or DLB; (2) investigate *in vivo* biomarkers using neuroimaging (such as structural MRI, functional MRI, PET) or fluid-based methods (CSF, plasma, or serum); (3) include sex-stratified results or analyses testing for sex-by-disease interactions within defined cognitive groups such as MCI, AD or DLB, and (4) be published in English in a peer-reviewed journal. Studies were excluded if they: focused exclusively on healthy or cognitively normal individuals, animal models, if they did not conduct sex-stratified or sex-interaction analyses within defined cognitive groups (i.e., MCI, AD, or DLB) or were review articles, editorials, or conference abstracts without peer-reviewed findings. All identified records were imported into Covidence systematic review software (Veritas Health Innovation, Melbourne, Australia) for screening and data management. EM conducted the initial title and abstract screening within the Covidence platform, followed by full-text assessment of potentially eligible articles against the inclusion and exclusion criteria.

## Results

### Study selection and characteristics

**Figure 1:**
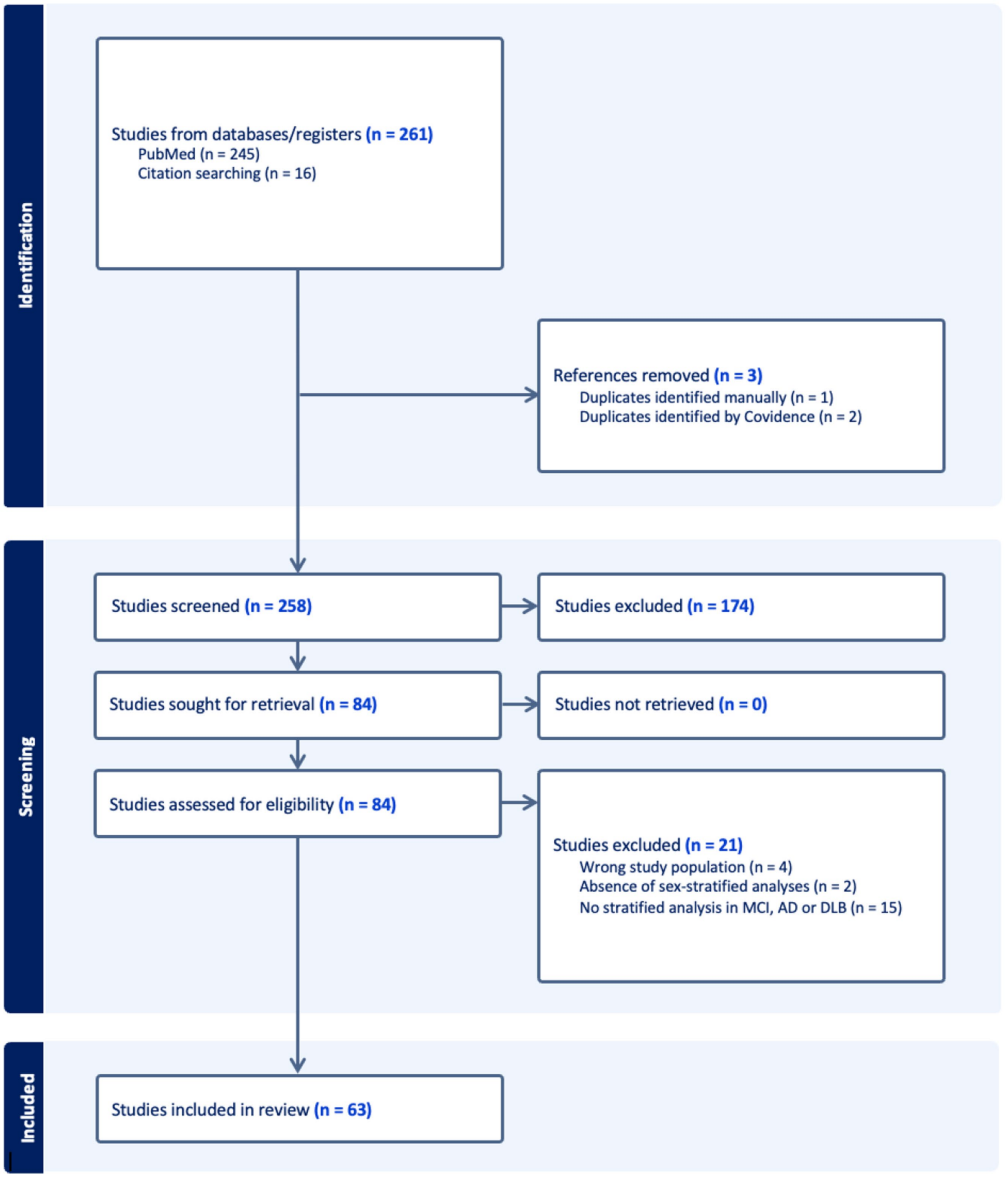
PRISMA flow diagram of study selection process. Flow diagram depicting the study selection process according to PRISMA guidelines. The final review included 63 studies that met all inclusion criteria. PRISMA, Preferred Reporting Items for Systematic Reviews and Meta-Analyses; MCI, Mild Cognitive Impairment; AD, Alzheimer’s Disease; DLB, Dementia with Lewy bodies.

**Figure 2:**
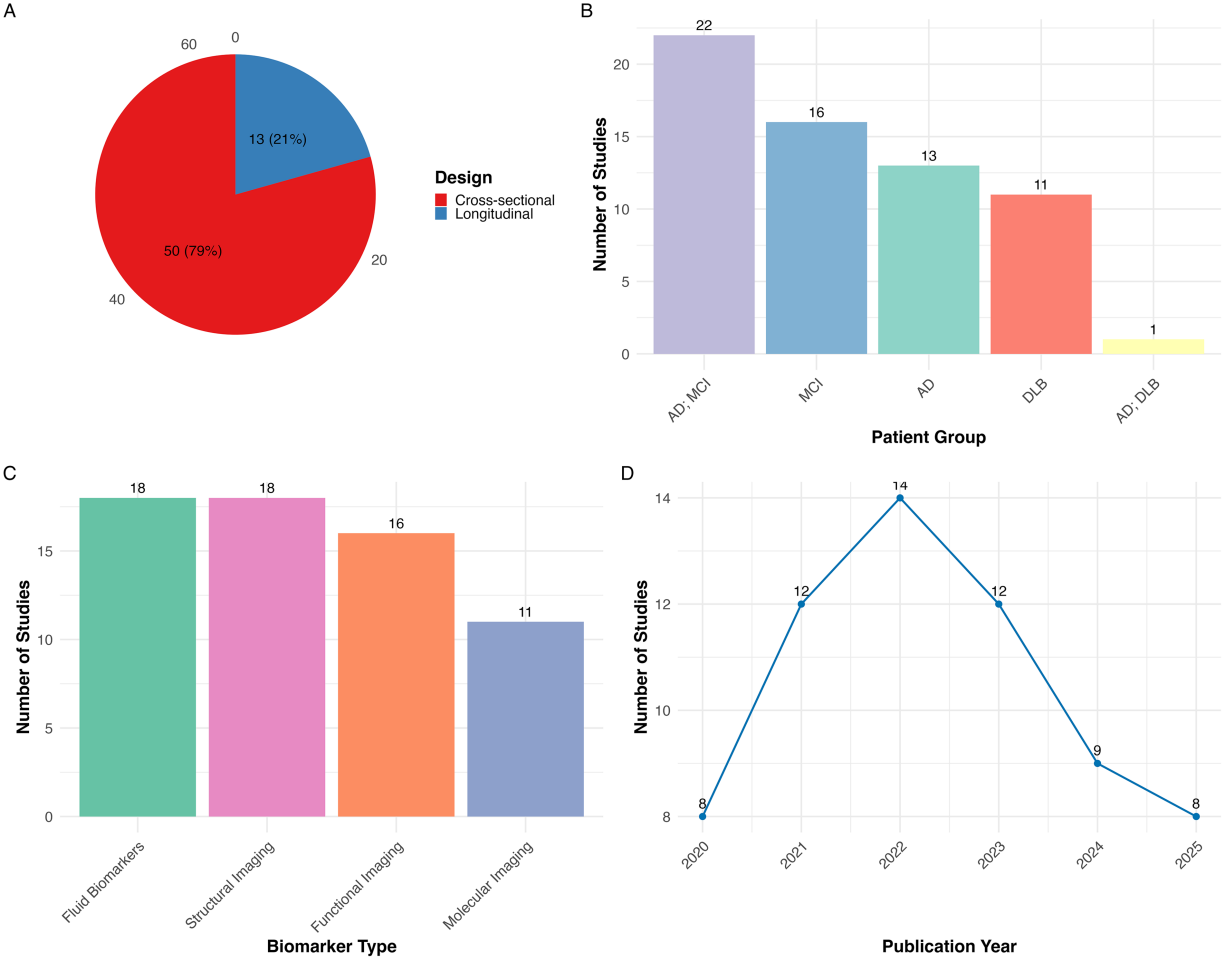
Summary of studies included in the systematic review of sex differences in dementia biomarkers (*N =* 63). **(A)** Distribution of studies by design showing that most studies were cross-sectional (*n =* 50, 78%) and the remainder were longitudinal (*n =* 13, 21%); **(B)** distribution of studies by patient group, indicating the number of studies conducted in AD+MCI, MCI, AD, DLB, AD+DLB; **(C)** distribution of studies by biomarker type, grouped into biofluid markers, structural imaging, functional imaging, and molecular imaging; **(D)** publication trend showing the annual number of studies from 2020 to 2025 with 2025 representing a partial year through May. AD, Alzheimer’s disease; MCI, Mild cognitive impairment; DLB, Dementia with Lewy bodies.

**Figure 3:**
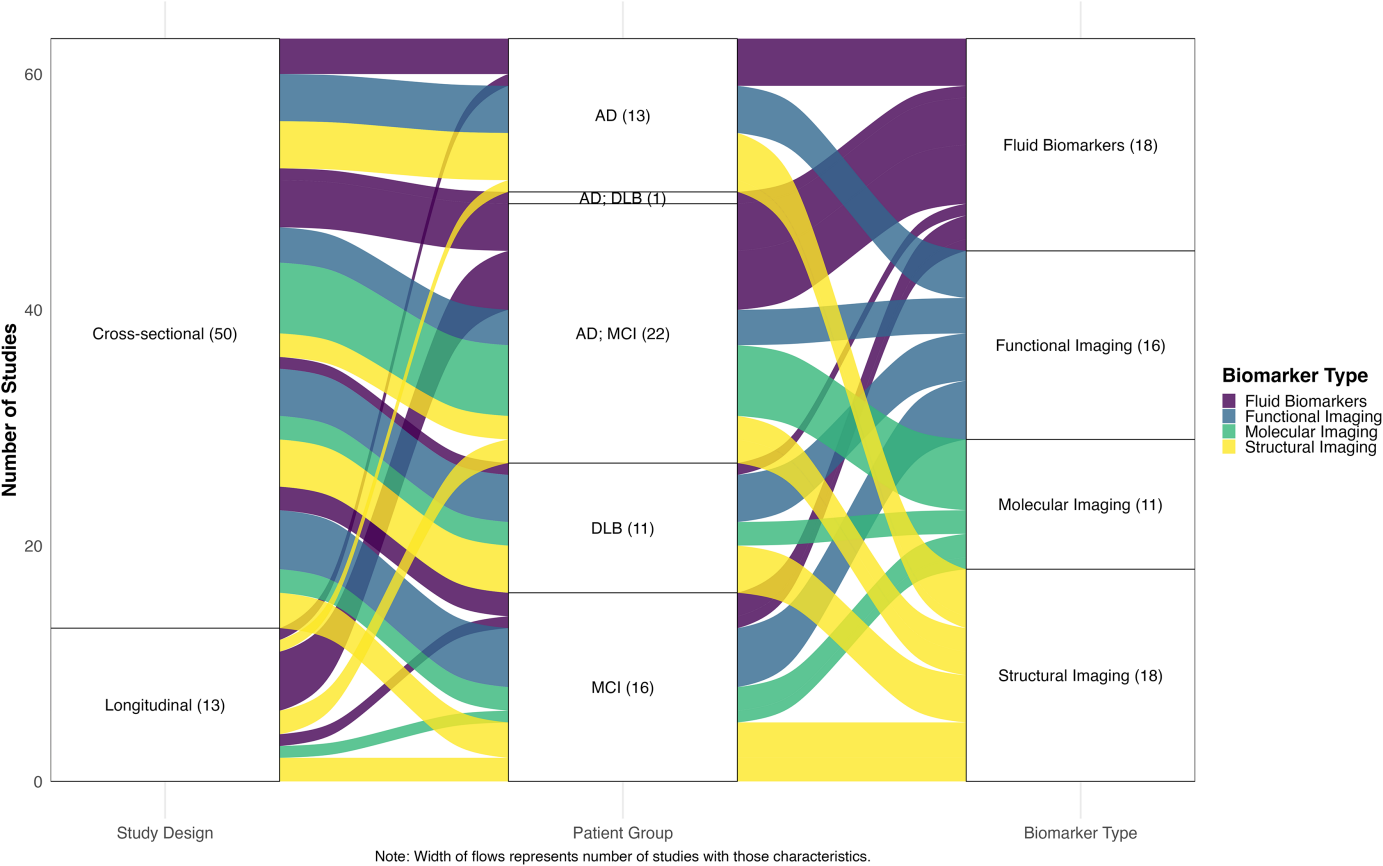
Alluvial plot of relationships between study designs, patient groups, and biomarker types. The width of the flows represents the number of studies with corresponding characteristics, illustrating the distribution and interconnections between methodological approaches across the included literature. AD, Alzheimer’s disease; MCI, mild cognitive impairment; DLB, Dementia with Lewy bodies.

A total of 261 records were imported for screening (245 from PubMed, 16 through citation searching). After removing 3 duplicates, 258 studies remained for screening. Of these, 174 were excluded during title and abstract screening for not meeting eligibility criteria. The remaining 84 full-text articles underwent a detailed eligibility assessment, after which 21 studies were excluded resulting in a final inclusion of 63 studies. The complete study selection process is illustrated in the PRISMA flow diagram (Figure 1). Of the included studies, 50 were cross-sectional and 13 were longitudinal in design. Regarding patient populations, 22 studies examined combined AD dementia and MCI due to AD cohorts, followed by 16 focused on MCI due to AD, 13 on AD dementia, 11 on DLB, and 1 study included both AD dementia and DLB groups. Studies were categorized into four biomarker modalities: Biofluid markers (*n =* 18), structural imaging (*n =* 18), functional imaging (*n =* 16), molecular imaging (*n =* 11). Figure 2 presents an overview of the included studies, whereas Figure 3 depicts an alluvial plot visualizing the relationships between study designs, patient populations, and distribution of biomarker modalities across the literature. A summary of the principal sex-related biomarker patterns across modalities is presented in Table 1.

**Table 1:** Summary of keysex differences in biomarker expression.

Biomarker domain	Women (↑/↓)	Men (↑/↓)
Biofluid markers	MCI/AD: ↑ CSF tau (p-tau181, t-tau), ↑ inflammatory markers, ↑ plasma GFAP	MCI/AD: ↑ plasma NfL and ↑ p-tau/NfL coupling
	DLB: ↓ CSF α-synuclein and Aβ42	
Structural MRI	MCI/AD: ↑ hippocampal volume early, ↓ cortical thickness later, ↑ WMH and ↑ correlation between WMH burden and cognition	MCI/AD: ↑ gradual cortical atrophy and ↑ correlation with disease progression
	DLB: ↑ more localized atrophy in the right entorhinal and olfactory cortices	DLB: ↑ atrophy in frontal/parietal regions especially at younger ages
Functional imaging	MCI/AD: earlier ↑ connectivity and metabolism followed by later ↓ decline; ↑ default mode hyperconnectivity; ↑ caudate connectivity with age	MCI/AD: more diffuse ↓ connectivity and metabolism across network
	DLB: localized ↓ metabolism; ↓ delta and ↑ posterior alpha EEG activity.	DLB: more extensive ↓ cholinergic network deficits
Molecular imaging (PET)	MCI/AD: ↑ amyloid and ↑ tau burden; ↑ tau network density and stronger associations with cognitive decline	DLB: ↑ dopaminergic deficits
	DLB: ↑ amyloid and tau burden	

Directional arrows (↑/↓) denote higher or lower biomarker levels, connectivity, or metabolic activity relative to the opposite sex. AD, Alzheimer’s disease; DLB, dementia with Lewy bodies; MCI, mild cognitive impairment; CSF, cerebrospinal fluid; NfL, neurofilament light chain; GFAP, glial fibrillary acidic protein; WMH, white matter hyperintensities; FDG-PET, fluorodeoxyglucose positron emission tomography.

### Biofluid markers

Eighteen studies examined sex differences in biofluid markers across the AD and DLB spectrum, revealing distinct patterns in cerebrospinal fluid (CSF) and blood-based markers of neurodegeneration, tau pathology, amyloid deposition, neuroinflammation, and metabolic dysfunction. While CSF is not routinely collected in most persons with AD or DLB, several studies have examined sex differences in CSF biofluids. Among APOE ε4 non-carriers, women exhibited significantly higher CSF phosphorylated tau (p-tau) and total tau (t-tau) concentrations in both MCI and AD dementia stages (Babapour Mofrad et al., 2020). This finding was corroborated by Ren et al. (2024), who found significantly higher t-tau concentrations in CSF among women with MCI (Ren et al., 2024). In DLB, CSF protein patterns differed markedly from AD. Women with DLB had significantly lower CSF *α*-synuclein and amyloid-β42 levels compared to men, accompanied by shorter symptom duration and more frequent hallucinations at diagnosis (van de Beek et al., 2022). Additional studies focused specifically on CSF neuroinflammatory markers revealed pronounced sex differences in immune responses too. The neuroinflammatory marker sTNFR2 showed women-specific associations with cognitive decline across the AD spectrum, with this relationship mediated through CSF p-tau181 levels (Bernier et al., 2022). Elevated baseline CSF TNF-α, interleukin-9, and IL-12p40 levels were associated with higher rates of conversion to MCI/AD, with women showing significantly shorter times to conversion when TNF-α levels were elevated (Contreras et al., 2022). Matrix metalloproteinase-9 (MMP-9) demonstrated sex-specific associations with CSF biomarkers: in men, MMP-9 was associated with higher CSF Aβ42, while in women, it correlated with higher CSF t-tau (Tsiknia et al., 2022b).

Blood biomarkers are more practical compared to CSF biomarkers. Plasma biomarker studies demonstrated sex differences in tau and amyloid pathology, metabolism, neurodegeneration, and neuroinflammation that varied across disease stages and showed distinct predictive value by sex. Plasma p-tau181 levels showed stronger associations with amyloid deposition in women with MCI compared to men; however, this relationship reversed in dementia stages, where plasma p-tau181 correlated with amyloid burden in men but not women (Tsiknia et al., 2022a). The p-tau181/Aβ42 ratio emerged as a stronger predictor of progression to AD, specifically in men with MCI (Berezuk et al., 2023). Liu et al. (2022) demonstrated that lower plasma Aβ42 was associated with faster memory decline in women but not men and that higher plasma Aβ42 levels predicted lower future AD risk specifically in women (Liu et al., 2022). Conversely, men displayed steeper increases in plasma pTau181, pTau231, and neurofilament light chain (Nfl) compared to women, along with steeper declines in brain volume and cognitive function (Joynes et al., 2025). In contrast, Labonte et al. (2025) found that higher plasma NfL levels predicted worsening dementia status only in women (Labonte et al., 2025). Women demonstrated significantly higher plasma GFAP levels compared to men in both DLB and AD, indicating enhanced astroglial activation (Bolsewig et al., 2024).

CSF neuroinflammatory markers revealed pronounced sex differences in immune responses to neurodegeneration. The neuroinflammatory marker sTNFR2 showed women-specific associations with cognitive decline, with this relationship mediated through CSF p-tau181 levels (Bernier et al., 2022). Elevated baseline levels of CSF inflammatory cytokines, including tumor necrosis factor *α* (TNF-α), interleukin-9 (IL-9), and IL-12p40, were also associated with higher rates of progression from cognitively normal to MCI or AD dementia. Women with elevated CSF TNF-α levels showed significantly shorter times to conversion, indicating sex-specific vulnerability to neuroinflammation (Contreras et al., 2022).

Sex-stratified metabolomic studies have begun to uncover distinct biochemical signatures in AD, revealing that men and women may follow divergent metabolic trajectories as the disease progresses. Men exhibited the most prominent metabolic alterations, particularly in phosphatidylcholines (e.g., PC ae C44:4, PC ae C42:3, PC ae C44:6) and amino acids (e.g., glycine, proline, glutamine), which were consistently reduced in AD compared to controls. These metabolites were enriched in glycerophospholipid metabolism, aminoacyl-tRNA biosynthesis, and arginine and proline metabolism pathways. In contrast, women-specific alterations were detected primarily in medium-chain acylcarnitines, including C10:2, C12:1, and C9, with reductions observed in both MCI and AD stages (Zarzar et al., 2022). Arnold et al. (2020) confirmed widespread sex-specific metabolic disruptions, identifying 53 metabolites with consistent sex differences across the MCI and AD spectrum (Arnold et al., 2020). Specifically, phosphatidylcholines and sphingomyelins were negatively associated with AD biomarkers in men but positively associated in women, suggesting divergent lipid dynamics. Similarly, acylcarnitines correlated with tau burden and cognitive decline in women but not in men, while amino acids showed stronger inverse associations with AD pathology in men. Additional plasma biomarkers demonstrated sex-specific patterns across disease stages. van Kruining et al. (2023) demonstrated that elevated plasma ceramide species C18:0, C24:1, and combined ceramide chain lengths were associated with MCI diagnosis exclusively in men (van Kruining et al., 2023). The clinical potential of sex-specific metabolic signatures was demonstrated by Xu et al. (2021), who found that vanillylmandelic acid (VMA) and tryptophan significantly improved AD prediction accuracy in women when combined with CSF markers (AUC increased from 0.884 to 0.955). This improvement was not observed in men, highlighting a sex-specific predictive advantage (Xu et al., 2021). In alignment with these findings, Kawakami et al. (2022) confirmed significant sex differences in serum lipid biomarkers, though these differences did not enhance diagnostic accuracy when analyzed separately (Kawakami et al., 2022). Notably, Chang et al. (2023) reported that the presence of APOE ε4 may override or modify observed sex-based differences in serum metabolites of AD patients (Chang et al., 2023).

Together, these studies highlight the multifaceted nature of sex differences in patterns of biofluid across the AD and DLB spectrum. Distinct patterns emerged across both CSF and plasma measures of tau, amyloid, neuroinflammation, neurodegeneration, and metabolism. Broadly, women tended to show greater vulnerability to tau pathology and neuroinflammatory processes, while men showed distinct amyloid dynamics and metabolic profiles. Notably, many of these associations were only detectable through sex-stratified or sex interaction analyses.

### Structural MRI

Structural brain MRI analyses employed diverse methods including automated volumetry, cortical thickness mapping, visual rating scales (e.g., Fazekas, MTA), and white matter hyperintensities (WMH) quantification as a marker of small-vessel cerebrovascular disease. Several studies revealed sex-specific patterns of neurodegeneration across the AD spectrum. Cortical thickness analyses revealed distinct sex-specific trajectories across disease stages. Women maintained greater cortical thickness across widespread regions during normal aging and early disease stages but exhibited steeper rates of cortical thinning from MCI to AD relative to men (Cieri et al., 2022). Extending these findings, Inguanzo et al. (2025) employed longitudinal atrophy subtype modeling in amyloid-positive AD individuals to demonstrate that women exhibited earlier hippocampal atrophy and a greater burden of white matter abnormalities than men (Inguanzo et al., 2025). Men showed more gradual, consistent thinning patterns across all disease stages. And, sex-related structural differences appear to be more pronounced in later AD stages compared to the prodromal MCI phase (Sauty and Durrleman, 2023). Women with established AD demonstrated higher gray matter volumes compared to men at equivalent disease stages while showing more severe gray matter atrophy during the MCI phase (Fernández et al., 2024). There are also regionally specific sex differences. For example, hippocampal analyses revealed sex differences across disease stages. Among individuals with amnestic MCI, women—particularly Hispanic and White non-Hispanic women—exhibited significantly larger baseline hippocampal volumes than men, independent of age, education, and APOE ε4 status (Garcia et al., 2024).

Unsupervised clustering analysis of MCI patients identified sex-specific disease subtypes. Women were more frequently classified into poor-prognosis subtypes characterized by smaller hippocampal and amygdalar volumes and greater cognitive deficits, while men more often clustered into better-prognosis subtypes with larger regional volumes (Katabathula et al., 2023). Similarly, Massetti et al. (2022) found that MRI-based prediction of MCI-to-AD conversion was significantly more accurate in women and younger individuals (Massetti et al., 2022). Longitudinal multimodal analyses revealed that men showed greater sensitivity to structural neuroimaging and CSF biomarkers—particularly hippocampal volume and tau/Aβ ratios—relative to women during the MCI to AD progression (Sarica et al., 2024).

Brain-age delta, an index of structural aging derived from MRI, also showed sex-specific biomarker associations. In MCI, brain-age delta in women was positively associated with plasma NfL, while in men, it correlated with WMH burden, suggesting that different neurobiological processes underlie aging trajectories in men versus women (Cumplido-Mayoral et al., 2023). Supporting this, Morrison et al. (2024) found that WMH burden was more strongly associated with cognitive impairment in women, and that women experienced more cognitive decline than men for equivalent WMH loads (Morrison et al., 2024). Supporting the notion of sex-dependent vulnerability across multiple structural domains, Kim et al. (2023) reported that women with AD exhibited lower connectivity strength within rich-club (i.e., densely interconnected hubs) and feeder networks (i.e., networks linking these central hubs to more peripheral regions), particularly involving the thalamus. Interestingly, thalamic connectivity was associated with cognition in men during prodromal AD and in women during AD dementia, suggesting stage- and sex-specific relevance of this network (Kim et al., 2023).

Using similar structural MRI approaches, others have also investigated sex differences in DLB, revealing distinct patterns of regional atrophy and age-related vulnerability. In a multicenter study combining data from the European-DLB consortium and Mayo Clinic, men exhibited widespread gray matter volume loss across frontal, temporal, and parietal regions, while women showed more localized atrophy in the right entorhinal and olfactory cortices (Oltra et al., 2023). Sex differences in atrophy were age-dependent, with men showing more severe neurodegeneration at younger ages than women with DLB, before diminishing after 75 years of age. Importantly, this regional atrophy correlated with cognitive impairment and visual hallucinations only amongst DLB men (Oltra et al., 2023). These findings were broadly aligned with visual rating analyses, which demonstrated that frontal atrophy was preferentially associated with DLB in men (Abdelnour et al., 2020). Using network analyses, Habich et al. (2024) revealed that normative sex differences in gray matter network organization were diminished in DLB. This network-level convergence aligns with the observation that sex differences in regional atrophy tend to diminish with increasing age. Sex differences in WMH burden were not observed in DLB (Ferreira et al., 2021).

In summary, sex differences in brain structure are evident across the AD spectrum and in DLB, encompassing both global and region-specific alterations that also manifest in prognostic subtypes, biomarker relationships, and network organization.

### Functional MRI

Data from a range of functional neuroimaging studies using functional MRI, FDG-PET, magnetoencephalography, and SPECT imaging have consistently revealed sex-dependent alterations in brain network organization, metabolic activity, and neurotransmitter pathways in both AD/MCI and DLB. One study found that women with MCI demonstrated significantly lower degree centrality (i.e., measure of how many direct network connections a brain region maintains), and global efficiency (i.e., the capacity of the brain network to efficiently integrate information across region) compared to men, with these sex differences diminishing during AD progression (Cieri et al., 2021). Age-related changes in caudate nodal strength, a graph-theoretical measure capturing how strongly a brain region is connected to the rest of the network, showed sex-specific patterns in MCI patients. Older age was associated with greater caudate connectivity strength in women but not in men (Yang et al., 2022). Complementing these findings, hippocampal connectivity to the precuneus cortex and brain stem was significantly stronger in men than women with MCI (Williamson et al., 2022). Additional network topology analyses revealed distinct sex-specific hyperactivity patterns: women showed default mode network-centered hyperactivity while men exhibited limbic system-centered hyperactivity across AD and MCI populations. Connectivity changes demonstrated stronger associations with network centrality in women (Wang et al., 2024). Resting-state fMRI studies in AD revealed complementary patterns, with men showing significantly lower connectivity strength within sensorimotor and attention networks, and network properties correlating with cognitive behavior specifically in men (Li et al., 2021).

Recent research is examining factors which may modulate the relation of sex with functional brain neuroimaging. One emerging area is the interaction between sex and genetic risk. Sex differences in connectivity has been found to interact with genetic risk factors. Indeed, Lin et al. (2020) demonstrated that among women MCI participants who were APOE ε4 carriers, fMRI showed reduced connectivity in multiple brain regions compared to non-carriers. Furthermore, a significant APOE-by-sex interaction was found, whereby cognitive scores were negatively associated with connectivity changes in men (Lin et al., 2020). Extending this line of work to later disease stages, Williamson et al. (2024) found that in AD dementia patients, intrahippocampal connectivity differences between APOE ε4 carriers and non-carriers only became apparent when analyses were stratified by sex (Williamson et al., 2024).

Metabolic brain changes evaluated using FDG-PET also showed sex-related trajectories. Longitudinal FDG-PET studies revealed sex-specific metabolic trajectories. In a South Korean AD cohort (*n =* 181), brain metabolic impairment at two-year follow-up was evident exclusively in women. Park et al. (2023) demonstrated in a longitudinal South Korean cohort (*n =* 181) of AD patients that brain metabolic impairment at two-year follow-up was evident only in women (Park et al., 2023). Similarly, Beheshti et al. (2021) found that while cognitively healthy women initially showed metabolically “younger” brains relative to men, this advantage was diminished in MCI AD (Beheshti et al., 2021). In early AD, higher serum VEGF levels were negatively associated with regional cerebral blood flow (rCBF) in the angular gyrus, with this effect driven primarily by men—suggesting a sex-specific link between angiogenesis and cerebral perfusion that may have therapeutic implications (Song et al., 2025).

Beyond metabolic and perfusion measures, electrophysiological and vascular imaging studies also revealed sex-dependent brain alterations. MCI patients showed sex-specific correlations between brain oscillations and CSF biomarkers. In men, lower CSF Aβ42 concentrations (reflecting greater amyloid burden) were associated with higher central–posterior theta power, a slower rhythm typically linked to memory encoding (Klimesch, 1999). However, in women, higher CSF t-tau levels were related to lower beta power, a faster oscillatory activity that has been associated with cognitive and emotional regulation (Chino-Vilca et al., 2022; Ray and Cole, 1985). Using dynamic contrast-enhanced MRI, blood–brain barrier permeability in the occipital cortex differed by sex in MCI, as permeability and education were predictive of cognitive scores exclusively in women (Moon et al., 2021).

While sex-related functional brain differences are well documented in MCI and AD, the extent to which they manifest in DLB has been less studied. Men with DLB exhibited more severe and diffuse disruptions in metabolic connectivity, particularly affecting cholinergic pathways including Ch4-perisylvian projections, while women with DLB showed more localized metabolic impairments (Caminiti et al., 2023). SPECT imaging revealed comparable striatal dopaminergic deficits between sexes. However, women demonstrated greater reductions in extrastriatal projections with more extensive long-distance disconnections between subcortical and cortical regions, while men exhibited more focal changes (Boccalini et al., 2023). The predominance of men was observed among cognitively unimpaired patients presenting with REM Sleep Behavior Disorder and reduced cardiac MIBG uptake (Fujishiro et al., 2021). Electrophysiological analyses further supported sex differences in DLB pathophysiology, as women with DLB demonstrated lower central-parietal delta activity and higher posterior alpha activity compared to men (Del Percio et al., 2025).

Collectively, these studies consistently demonstrated sex-dependent differences in network connectivity, metabolic activity as well as and neurophysiological signatures in AD and DLB. Women tended to showed earlier functional advantages followed by steeper decline, while men exhibited more gradual but widespread functional impairments. Moreover, APOE ε4 modified these patterns differentially by sex.

### Molecular imaging biomarkers of AD

Molecular neuroimaging is a rapidly evolving field. Recent studies using brain PET radiotracers for amyloid and tau demonstrated significant sex differences both with respect to the severity of pathological protein accumulation and distribution patterns. In the largest “real-world” amyloid PET datasets to date, Abu Raya et al. (2025) analyzed 10,361 patients with cognitive impairment—including both MCI and dementia—and found that women had significantly higher amyloid PET positivity rates and greater amyloid burden than men, independent of age, comorbidities, and other demographic and clinical covariates (Abu Raya et al., 2025). Women with MCI or AD exhibited significantly greater amyloid (PIB-PET) and tau (Flortaucipir-PET) accumulation in AD-relevant brain regions than men, despite being clinically comparable in terms of cognition (MMSE, CDR-Sum of Boxes, episodic memory) and cortical thickness (Edwards et al., 2021). Consistent with these findings, Buckley et al. (2020) found that MCI women exhibited higher tau-PET signal than men across widespread cortical regions—particularly among *β*-amyloid–positive women—and that elevated tau burden predicted faster cognitive decline in women compared to men, suggesting greater tau vulnerability and accelerated disease progression in women (Buckley et al., 2020).

Sex differences in tau burden were pronounced during the MCI phase, with tau deposition correlating more strongly with verbal memory performance in women (Banks et al., 2021). Men with prodromal AD showed significantly lower tau burden compared to women (Digma et al., 2020). Sex-by-APOE ε4 interactions were observed across multiple cohorts. Women who are APOE ε4 carriers showed higher tau burden in the medial temporal lobe regions compared to men carriers (Wang et al., 2021). This pattern was further substantiated by Yan et al. (2021), who found that male APOE ε4 homozygotes showed significantly higher tau deposition than male heterozygotes, whereas female APOE ε4 heterozygotes already showed greater tau deposition across multiple regions, suggesting a sex-dependent dose–response relationship between APOE ε4 and tau pathology (Yan et al., 2021).

Beyond regional analyses, network-level investigations revealed distinct sex-specific patterns of tau spread. Shokouhi et al. (2020) demonstrated that tau networks amongst women were characterized by significantly greater network density and more numerous direct regional connections compared to men, with these differences most evident at the MCI stage of AD (Shokouhi et al., 2020). The functional implications of these sex-specific tau patterns extend to neurochemical systems as well. Wang et al. (2025) demonstrated that hippocampal mGluR5 receptor availability was negatively associated with tau deposition and cognitive associations were only present in women with AD dementia, where tau deposition mediated the receptor-cognition relationship (Wang et al., 2025). In DLB, women were more likely to exhibit concurrent amyloid and tau pathology compared to men in a multicenter study of 417 patients (Ferreira et al., 2020). In summary, there are robust sex differences in both the burden and spatial distribution of amyloid and tau pathology, with women often showing higher pathological burden and stronger cognitive associations than men in both AD dementia and DLB.

## Discussion

Our review demonstrates that biological sex has strong influences on biomarker expression across AD dementia and DLB, revealing three broad patterns with potential bearing for precision medicine and sex-informed therapeutic approaches. In the following section, we interpret the broad themes emerging from this review, outline several limitations of current research, and propose future research priorities for advancing our mechanistic understanding of sex differences in AD and DLB.

Women exhibit a distinctive pattern of early structural and metabolic advantages followed by steeper decline trajectories during disease progression. Structural neuroimaging studies demonstrate that women maintain larger hippocampal volumes and greater cortical thickness during normal aging and early disease stages including MCI (Cieri et al., 2022; Garcia et al., 2024), yet exhibit accelerated atrophy rates as pathological processes advance and dementia becomes manifest (Sauty and Durrleman, 2023). This structural pattern extends to metabolic function, where women initially demonstrate metabolically younger-appearing brains but experience more aggressive decline in MCI and AD dementia stages compared to a more gradual decline in FDG signal as observed in men (Beheshti et al., 2021; Park et al., 2023). Nonetheless, some studies report differing trajectories—for example, recent studies have shown that women with amyloid-positive AD have slower cognitive decline than men despite exhibiting earlier hippocampal atrophy and a greater burden of white matter abnormalities (Inguanzo et al., 2025) and men have demonstrated steeper increases in plasma pTau181, pTau231, and NFL (Joynes et al., 2025), underscoring the complexity and heterogeneity of sex effects across cohorts and *in vivo* biomarkers. It is likely that other factors, including some that researchers have yet to define and measure, play a role in the complex relation of sex to neurodegenerative disease expression.

The preservation of hippocampal volumes and cortical thickness during early disease stages may serve to relatively preserve episodic memory function while other cognitive domains become impaired, potentially partially explaining why women tend to present with impairments in verbal memory and semantic fluency rather than episodic memory deficits in early cognitive decline (Aggarwal and Mielke, 2023). An alternative or complementary explanation is that women may start from a higher baseline performance in these domains earlier in life, providing a cognitive reserve that temporarily offsets the impact of neuropathology. Such selective preservation may initially mask the clinical impact of accumulating tau pathology and could have important implications for detection and accurate early diagnosis, as they suggest that conventional biomarker thresholds may systematically underestimate disease burden in women during early stages while simultaneously overestimating progression risk in men. In addition, the stronger biomarker-clinical correlations that were documented in women across multiple biomarkers and modalities (Banks et al., 2021; Burke et al., 2019; Morrison et al., 2024; Tsiknia et al., 2022a; Yang et al., 2022) suggest differential trajectories, indicating that interpretation of biomarkers may require sex-specific frameworks to optimize diagnostic accuracy and therapeutic timing. Indeed, prediction models for MCI-to-AD conversion demonstrate significantly higher accuracy in women compared to men (Massetti et al., 2022) and hippocampal volume changes are more predictive of AD progression in women than men (Burke et al., 2019).

The literature also revealed sex-specific patterns of vulnerability that differ between AD and DLB, suggesting that disease-specific pathological processes (i.e., aggregation of amyloid and tau versus *α*-synuclein) may interact with biological sex. In AD, women demonstrate consistently higher tau burden across multiple brain regions (Buckley et al., 2019; Digma et al., 2020; Edwards et al., 2021), with the most pronounced sex differences appearing during the MCI stage, as tau deposition shows stronger associations with verbal memory performance in women (Banks et al., 2021). This extends beyond regional tau accumulation to broader network-level differences, with women showing greater tau network density and more extensive regional connections (Shokouhi et al., 2020). Women also demonstrate higher CSF phosphorylated and total tau levels during MCI and AD stages (Babapour Mofrad et al., 2020; Ren et al., 2024). In contrast, DLB demonstrates a vulnerability pattern in men characterized by widespread structural damage and severe metabolic connectivity disruptions, particularly affecting cholinergic pathways. Men with DLB exhibit more extensive gray matter atrophy across frontal, temporal, and parietal regions, with these structural changes appearing to correlate with cognitive impairment and visual hallucinations exclusively in men (Abdelnour et al., 2020; Oltra et al., 2023). Men also demonstrated more severe and diffuse disruptions in metabolic connectivity, particularly involving cholinergic pathways (Caminiti et al., 2023). However, women with DLB are more likely to exhibit concurrent amyloid and tau pathology (Diaz-Galvan et al., 2023; Ferreira et al., 2020) and show lower CSF α-synuclein and amyloid-β42 levels, shorter symptom duration, and more frequent hallucinations at diagnosis (van de Beek et al., 2022). This mixed pathology profile is corroborated by prior autopsy data demonstrating that despite having higher regional Lewy body, neurofibrillary tangle, and senile plaque burdens across multiple brain regions, women with pathologically-confirmed DLB remain significantly less likely to receive clinical diagnoses compared to men (Bayram et al., 2025; Nelson et al., 2010). This pattern suggests a more aggressive disease phenotype in women despite less extensive structural damage (Oltra et al., 2023), highlighting both the complexity of sex-disease interactions and potential diagnostic bias that may systematically underrecognize DLB in women.

The vulnerability to tau pathology in AD described previously among women may – at least in part – be further amplified by genetic risk factors. The APOE ε4 allele demonstrates sex-specific effects that exacerbate women’s susceptibility to tau accumulation and neurodegeneration. For instance, women who are APOE ε4 carriers exhibit accelerated tau accumulation in medial temporal regions, higher CSF tau levels, and stronger associations between plasma biomarkers and brain pathology compared to men (Liu et al., 2019; Wang et al., 2021). We speculate that the more severe tau pathology observed in women who are APOE ε4 carriers may mediate downstream functional consequences, as connectivity analyses revealed widespread network impairments, particularly affecting women who are carriers (Lin et al., 2020), though longitudinal studies with appropriate statistical models would be needed to define causal pathways. The dose–response relationship between APOE ε4 and pathological burden or neurodegenerative outcomes also appears to differ by sex. For instance, women who are heterozygotes demonstrate more pronounced tau effects than men who are heterozygotes, while men who are homozygotes show dose-dependent increases in tau deposition not observed in women who are homozygotes (Yan et al., 2021). This heightened vulnerability to APOE ε4 among women is supported by epidemiological evidence showing that women with at least one copy of APOE ε4 exhibit greater risk and faster cognitive decline relative to men (Neu et al., 2017). Furthermore, women with APOE ε4 show higher rates of conversion from MCI to AD compared to non-carrier women or men (Beydoun et al., 2012). The biological basis for this vulnerability in women may involve both developmental and adult hormonal influences on brain structure and function (R. Li and Singh, 2014). Indeed, endocrinological factors and particularly sex steroid hormones, have been associated with AD onset and progression, with age-related depletion of estrogens in women and androgens in men resulting in loss of neuroprotective effects (Rosario et al., 2011).

Several limitations in this review should be acknowledged. First, this review relied on a single database (PubMed) and screening was conducted by one reviewer, which may have introduced selection bias by potentially omitting eligible studies indexed in other databases or by limiting validation of study inclusion decisions. However, this risk was partly mitigated as we performed citation searching of reference lists from included articles to identify additional eligible studies where possible. There is still scarce data on how sex differences in biomarkers evolve longitudinally, particularly following the same sample through the transition from MCI to AD or prodromal *α*-synucleinopathies to DLB. Additionally, the underlying biological mechanisms driving these sex differences remain largely unexplored, including the roles of hormonal factors, hormone replacement therapy use, estrogen-mediated neuroprotective mechanisms, and their complex interactions with genetic risk factors such as APOE ε4 and others. Furthermore, the lack of large-scale, multi-center studies may limit the generalizability of findings across diverse populations and healthcare settings, a challenge that is compounded by the small sample sizes available for direct comparisons between women and men in many studies. This limitation is particularly more acute for biomarkers that are costly, technically demanding, or invasive— all of which factors that may limit scalability and further restrict the feasibility of adequately powered, sex-specific studies in AD and DLB. Such constraints not only diminish statistical power but also increase the risk of spurious findings, making it more challenging to detect subtle yet potentially meaningful sex-specific effects. They also restrict the generalizability of results across diverse populations and healthcare settings and limit our understanding of how sex differences manifest across different demographic and geographic contexts. To this end, ongoing initiatives that pool data across large consortiums such as the National Alzheimer’s Coordinating Center (NACC)^1^ and the European-DLB consortium^2^ offer promising opportunities to address these limitations and enable more robust analyses of sex differences in adequately powered, multi-site cohorts. In parallel, the growing awareness in the research community and development of data harmonization methods, such as ComBat and similar statistical approaches (Fortin et al., 2017), further facilitate these multi-center efforts by addressing site-specific technical variations and enabling more reliable cross-site comparisons.

Our review highlights the need for prospective, sex-stratified studies to better understand how biological sex shapes biomarker expression and disease progression. Firstly, longitudinal studies are well-suited to define the optimal timing and sequencing of different biomarker modalities for women and men, with particular attention to identifying the narrow therapeutic windows that appear to precede accelerated decline in women (Beheshti et al., 2021; Park et al., 2023). More sophisticated neuroimaging techniques, such as Neurite Orientation Dispersion and Density Imaging (NODDI), may yield deeper insights into microstructural sex-related differences (Mak et al., 2025b; Zhang et al., 2012), while emerging α-synuclein biomarkers, including CSF seed amplification assays and other synuclein-based fluid biomarkers may reveal sex-specific patterns in Lewy body pathology (Mak et al., 2025a). Building on the promising findings from single-modality studies, future multi-modal investigations could elucidate how metabolic differences interact with genetic risk factors, neuroimaging profiles, and longitudinal clinical trajectories to influence sex-specific disease mechanisms too. Importantly, the sex-related *in vivo* biomarker patterns identified in this review would need validation through larger, well-characterized autopsy series and to determine whether imaging vs. pathological correlations differ between sexes. Additionally, research examining sex differences in response to emerging disease-modifying therapies, including anti-amyloid, anti-tau, and anti-α-synuclein treatments, will be essential for optimizing personalized treatment strategies. In the long term, we recommend that clinical studies and trials should be designed from the outset with adequate power to detect sex-specific treatment effects, as distinct pathological vulnerabilities in men and women may translate into differential therapeutic responses. As described, sex-stratified analytical approaches have already shown promise for improving diagnostic accuracy and informing personalized interventions (Massetti et al., 2022; Tsiknia et al., 2022a; Wang et al., 2021).

## Conclusion

Our review demonstrates that biological sex shapes biomarker expression in AD dementia and DLB, and poses challenges to current diagnostic and therapeutic paradigms that are largely agnostic to sex differences. Understanding these sex-specific mechanisms offers novel opportunities to improve early detection, refine therapeutic timing, and optimize personalized interventions for men and women. To these ends, the growing emphasis on sex-stratified analyses by major funding agencies such as the NIH and Alzheimer’s Association is anticipated to help address knowledge gaps in both AD and DLB research and advance our understanding of sex-specific mechanisms across neurodegenerative conditions. Ultimately, embracing biological sex as a fundamental determinant of neurodegenerative disease may transform our approach to preventing, diagnosing, and treating these conditions.

## Data Availability

The original contributions presented in the study are included in the article/supplementary material, further inquiries can be directed to the corresponding author.

## Footnotes

1
www.naccdata.org

2
www.e-dlb.com
